# Cases of Inversio Uteri

**Published:** 1857-03

**Authors:** P. G. Bertolet

**Affiliations:** Oley, Berks Co., Pa.


### ART. VII.—*Cases of Inversio Uteri.* By P. G. BERTOLET, of Oley, Berks Co., Pa.

CASE 1st.—Mrs. D. K., æt. 40, had *complete inversion of the uterus* of the *chronic* variety, *reducible* (using the term as applied by Dr. Radford, of Manchester). I saw this patient first on Sept. 21st, 1852, while she was laboring under typhoid fever, and from which she died in six days after. She had been afflicted with the loathsome affection, first mentioned, for nearly eight years. The womb would occasionally become inverted, in spite of all contrivances and applications of bandages and uterine supporters. The present inversion, I was told, came on while she was confined to bed in the recumbent position. It seemed as if the uterine fibres had acquired an abnormal habit of inverting. From the patient's general debility, there was also less power of resistance in the other parts. The organ was not much indurated, presented a livid hue, and was covered with dry epithelial scales. Owing to her state of debility, she could not now endure to have the organ completely replaced, and this, no doubt, lent considerable influence toward the speedy dissolution.

CASE 2d.—Mrs. D. Y. P. was placed under my professional care in the spring of 1849. June 6th, she was delivered of a healthy female child. Previously she had had two children, who are yet living, but she aborted preceding her last confinement, which caused her friends some anxiety in regard to her present condition. But on this occasion she went to her full time, and had an easy, but somewhat tardy delivery, and a speedy recovery.

Sept. 12th, 1850. Aborted with a male fœtus of about five months. Her getting up was apparently good.

July 4th, 1851. Aborted with another male fœtus of four months.

May 3d, 1852. Aborted.

October 15th, 1854. Again aborted; but I not being at home just at the moment, my friend, Dr. Herbst, was called in, and when I arrived, he had already removed the child; but informed me that the secundines were retained by a large tumor in the mouth of the womb and upper part of the vagina, which might prove to be another ovum. Upon examination, I was led to confirm his opinion. The woman at the same time had bearing-down pains. The tumor felt rather more tense to the touch than the membranes do ordinarily, but this was thought might be owing to the early period of gestation, since they are then generally found firmer and more resisting.

After waiting some time, the tumor came down, but, to our sorrow, we found it to be the womb, which now became *completely inverted.* The placenta was peeled off, and but little hemorrhage supervened. It was with the utmost difficulty that the organ was got back, and the fundus did not maintain its normal position, even after we had succeeded in replacing it.

Acute metritis supervened, and the woman died the fifth day after the occurrence.

*Remark.*—The frequent abortions might, perhaps, be assigned as the cause of the inversion in this case, yet it seems to me a question, whether the cause of the frequent abortions was not due to a pre-existing depression of the fundus, or *partial* inversion, as Dr. Ashwell terms it.

CASE 3d.—Mrs. J. K., æt. 24, was confined with her first child, Oct. 24th, 1856. She sent for me at the break of day, and upon employing the *touch*, I found the os tincæ dilated to about the size of a twenty-five cent piece. The pains recurred regularly, but at long intervals; otherwise she was doing well. Having several other patients in the vicinity, I left her for a few hours. On my return, at 10 A.M., I found labor progressing; and at 12 M., she was delivered of a fine female child. After the latter was removed, the placenta was found on examination, in the mouth of the womb, and by making gentle traction on the cord, came readily away. A considerable gush of blood followed, causing her to feel faint; but after lowering her pillows, she soon rallied, and the hemorrhage ceased. When I left her, she expressed herself as feeling pretty comfortable.

October 25th. Called early this morning. In order to maintain her quietude as much as possible, I drew off her water. Doing pretty well, feels only weak, which I attributed to the loss of blood yesterday.

I saw her daily until the seventh day after delivery, when she was doing apparently as well as could be expected. Passed water with ease, lochia moderate, secretion of milk abundant, appetite good, and strength so much regained that she could sit up and have her bed made. I left her, with advice to keep herself easy, and not to get out of bed too soon.

On the morning of Nov. 2d, the ninth day after delivery, I was sent for in great haste, the messenger stating that the patient had suddenly become very poorly, and that something was coming away from her. I found the woman lying upon her back, thighs drawn up, countenance pale and expressive of intense suffering, pulse very small and quick. A tumor, of about the size of my two fists, was found between her thighs, which proved to be the womb in a state of *complete inversion*. According to her statement, this state of things came on suddenly this morning, while on the close-stool for the purpose of voiding her water, in which she experienced some difficulty, and on making bearing-down efforts, "all at once everything gave way."

The womb was hard, and its vessels filled with blood, from the contraction of the os tincæ preventing its reflux; and but very slightly yielding upon pressure. There was no bleeding, save a little oozing, and the surface, when wiped clean, presented a dark red or livid hue, particularly that portion to which the placenta had been attached, which could readily be distinguished from its more rugose aspect and darker color.

The bladder was first emptied with a silver male catheter (it being impracticable to introduce any other). The womb was then enveloped in fine soft linen, and gently pressed between my two hands, in order to force out of its tissue as much blood as possible, and thereby diminish its size. In the course of twenty minutes, it became so reduced, that by a little upward pressure it passed again into the vagina. I next endeavored to indent the fundus with my two forefingers, previously well-wadded and anointed. In this I found great difficulty, but after some time succeeded, but could not carry it through the os uteri. In order to gain space, I wadded the handle of a spoon, and placed it in the position of my fingers, which I now withdrew, and continued gentle pressure; but this failed. The parts were again well anointed, and the hand introduced into the vagina, and as before, with my two fingers, I made steady pressure and continued it, found the os gradually dilating, and after one and a half hour's patient perseverance, had the satisfaction of replacing the organ in its natural position. The T bandage was then applied, and that of the abdomen removed. The woman soon expressed herself as feeling more comfortable. The operation gave her some pain, yet she bore it quite heroically, considering her condition. I did not feel justified in this case to make use of anæsthetic agents.

Nov. 3d, 8 A.M., tolerably comfortable, only weak; womb remained in its place; passed water; lochia moderate; some secretion of milk; appetite good; tongue clean; pulse 100 per minute; slept some during the night. Ordered the use of compound tr. bark, and an opiate at bed-time.

4th. No pain, but passed a restless night; abdomen somewhat tympanitic; bowels not moved since lavement, some six or seven days after accouchement; no lochia to-day; tongue thinly coated but not dry; some thirst; passed water. Directed the continuance of the bark with spts. nit. dulc., and $\frac{1}{4}$ gr. morphia sulph., at bed-time, to be repeated during the night if the first dose failed to afford rest.

5th. Very weak; tongue more coated; thirst; pulse weak and frequent; lochia very scant; womb upon examination found in its proper position. The bowels were ordered to be moved with oil and glysters.

6th. Oil operated; feels better this morning; stronger; some appetite; secretes a little milk; tongue less coated; pulse 90 per minute; fuller and stronger; rested comfortably during the night.

From this time forward she continued to improve steadily; and nothing untoward occurred except a somewhat painful œdema of her right leg, which prevented locomotion for several weeks. Several months have now elapsed, and I am happy to learn that she has recovered perfectly.

NOTE.—The subject of this case went through her term of pregnancy with regularity. She saw the last of her catamenia January 15th, '56. Is now married three years; was previous to pregnancy much troubled with dysmenorrhœa, which may probably have prevented earlier conception. Otherwise she always enjoyed good health, and is of a hale and robust constitution.

